# How Relative Age Effects Associate with Football Players’ Market Values: Indicators of Losing Talent and Wasting Money

**DOI:** 10.3390/sports9070099

**Published:** 2021-07-09

**Authors:** Michael Romann, Marie Javet, Stephen Cobley, Dennis-Peter Born

**Affiliations:** 1 Department for Elite Sport, Swiss Federal Institute of Sport Magglingen, 2532 Magglingen, Switzerland; Marie.Javet@baspo.admin.ch (M.J.); dennis.born@swiss-aquatics.ch (D.-P.B.); 2 Faculty of Health Science, University of Sydney, Lidcombe, NSW 2141, Australia; stephen.cobley@sydney.edu.au; 3 Swiss Swimming Federation, 3063 Berne, Switzerland

**Keywords:** talent selection, talent development, return of investment, market value, drafts

## Abstract

Background: In football, annual age-group categorization leads to relative age effects (RAEs) in talent development. Given such trends, relative age may also associate with market values. This study analyzed the relationship between RAEs and market values of youth players. Methods: Age category, birthdate, and market values of 11,738 youth male football players were obtained from the “transfermarkt.de” database, which delivers a good proxy for real market values. RAEs were calculated using odds ratios (OR) with 95% confidence intervals (95%CI). Results: Significant RAEs were found across all age-groups (*p* < 0.05). The largest RAEs occurred in U18 players (Q1 [relatively older] v Q4 [relatively younger] OR = 3.1) ORs decreased with age category, i.e., U19 (2.7), U20 (2.6), U21 (2.4), U22 (2.2), and U23 (1.8). At U19s, Q1 players were associated with significantly higher market values than Q4 players. However, by U21, U22, and U23 RAEs were inversed, with correspondingly higher market values for Q4 players apparent. While large typical RAEs for all playing positions was observed in younger age categories (U18–U20), inversed RAEs were only evident for defenders (small-medium) and for strikers (medium-large) in U21–U23 (not goalkeepers and midfielders). Conclusions: Assuming an equal distribution of football talent exists across annual cohorts, results indicate the selection and market value of young professional players is dynamic. Findings suggest a potential biased selection, and undervaluing of Q4 players in younger age groups, as their representation and market value increased over time. By contrast, the changing representations and market values of Q1 players suggest initial overvaluing in performance and monetary terms. Therefore, this inefficient talent selection and the accompanying waste of money should be improved.

## 1. Introduction

During childhood and adolescence, young football players are categorized by annual age groups. However, the chronological age gap of up to 12 months between players born in early (January) and late (December) in the year leads to substantial differences in performance and biased talent selection decisions [1,2]. The result of participation or selection bias, specifically the overrepresentation of chronologically older soccer players within one age category, is called relative age effects (RAEs). RAEs have been shown to affect talent development systems in a wide range of team and individual sports, e.g., ice hockey, football, swimming, tennis, in both females and males from 4 years of age to adulthood [2,3]. Relatively older children within annual cohorts are more likely to be selected in talent development teams, with selection commensurate with additional training, and access to higher quality coaching likely leading to accumulated performance advantages [4,5]. By contrast, the relatively younger children are underrepresented, are less likely to be selected to talent development systems, and are more likely to withdraw from the sport [6,7,8]. Interestingly, research has subsequently shown how relatively younger players, who are selected for a talent development system, actually have a greater chance of becoming a professional player than their relatively older counterparts [9]. Such observations have become synonymous with the proposition of a “underdog hypothesis” [10]. In talent development contexts, late-born players have been shown to be more likely to achieve senior professional status, as they may benefit more from competitive play with their older counterparts [10,11,12]; that said multiple factors and processes may contribute to the outcome. Furthermore, a study of German professional soccer players has shown that players born late (Q4) had systematically higher wages than their fellow Q1 players [13].

For football clubs, the capability to accurately identify athletic potential, and recruit potential, in the early stages of development has several organisational benefits [14]. Given how athletic talent can influence team achievements, being able to secure athletic potential can have performance benefit [15]. That said, research which examines the hiring decisions in professional sports, recognized the difficulty of being able to accurately identify youthful talent, which may lead to future performance productivity [16].

In addition to the traditional assessment methods of talent scouts, fans and football experts have established a large online community called “transfermarkt.de”. Transfermarkt.de assesses the market value of professional footballer players at an age range from U15 to retirement. The community has become the main source for reporting on market values [17,18]. From an economic perspective, the aim of many professional football clubs is to buy undervalued players to achieve both higher performance and higher returns on investment [18]. Moreover, a rapidly growing body of literature emphasizes the importance of collective judgements for assessing actual and future values [17,19]. Recent studies showed that the variance of actual transfer fees paid (for players) in the German Bundesliga can almost entirely be explained (R^2^ = 0.90) by the market values reported on transfermarkt.de [17]. Current literature suggests that player market values on transfermarkt.de are good proxy estimate indicators of current as well as future players’ real market values and will, therefore, play an increasing role in talent recruitment, sports economics and talent development [17,19].

Given the relevance of RAEs and market values for professional soccer clubs this study had two objectives. The first objective was to identify the presence of (changing) RAEs in professionally contracted players across the developmental to professional years (e.g., 18–23 years of age). The second objective was to assess the relationship between RAEs and player market values (as indicated on Transfermarkt) and whether age-group and playing position moderated the relationship.

## 2. Materials and Methods

### 2.1. Participants

Participants were n = 11,738 players included in this study. Inclusion criteria were 2000 players with the highest market valuein every age categories from U18 to U23. In the U18 category all 1738 listed players were analysed (Table 1).

Data were provided by the owner of the open-source football database transfermarkt.de, with permission to anonymously analyse and publish the results. All data were extracted on 17 July 2020 and included current data of players age, height, market value, club and nationality. The website provides independent estimates of players’ market value and is regularly updated (last update in March 2020) by more than 190,000 professional and non-professional individuals with the approval of Transfermarkt.de experts [17,20]. Transfermarkt.de has been used in several previous studies [20,21,22,23], and has been shown to be a valid and useful database for game performance indicators and market values [17]. Data exported for this study included birthdate, market value, nationality, club and playing position. The study was pre-approved by the institutional review board of the Swiss Federal Institute of Sport Magglingen (Reg.-Nr. HLP-2021-131) and is in accordance with the Declaration of Helsinki.

### 2.2. Procedures and Data Analysis

The cut-off date for age group selections in international football in all countries and according to FIFA rules is 1 January. Players were categorized into four relative age quarters (Q) and two relative age semesters (S) according to their birth month, independently of birth year (i.e., S1 = January to June; S2 = July to December and Q1 = January to March; Q2 = April to June; Q3 = July to September; and Q4 = October to December). Due to the multi-nation sample (n = 152) within the current investigation, potential national differences in birth rates per month could not be taken into consideration which has to be considered as a limitation. Therefore, equal distribution of births across all months and years was assumed for the expected birth distribution of the general population [1,5]. The following age categories were analysed for their relative age distributions: U18 to U23. RAEs were calculated using odds ratios (OR; Q1 vs. Q4) with 95% confidence intervals (95%CI). The OR was interpreted as an effect size as follows: we assumed a significant RAE if the CI did not include 1 and interpreted 1.00 ≤ OR < 1.22, 1.22 ≤ OR < 1.86, 1.86 ≤ OR < 3.00, and OR ≥ 3.00, as negligible, small, medium and large, respectively [24]. If the OR was <1 and the CI did not include 1, this finding was interpreted as a significant inverse RAE. Inverse ORs < 0.33 (1/3), 0.33 ≤ OR < 0.53 (1/1.86), 0.53 ≤ OR < 0.81, 0.81 ≤ OR < 1.0 were, respectively, interpreted as large, medium, small, and negligible. Market values were extracted in €, playing positions were categorized as goalkeepers, defenders (central and outside), midfielders (central and outside) and strikers. Using these data mean market values per age category and Q were calculated using crosstabulations. In a second step the difference of observed and expected market values (Δ) were calculated. Observed market values were the sum of the market values of all players per age category and per Q. Estimated market values were calculated in the same way, but with the assumption of an equal distribution of players per Q. For instance, if the expected number of players in each Q is 500, the observed number in Q4 is 400 and the mean market value of the Q4players of the age category is 1,000,000€, the calculated Δ is 400 − 500 = −100 × 1,000,000€ = −100,000,000€.

## 3. Results

Distribution of players per Q with 95% CI are illustrated in Table 2. There were medium RAEs in the U18 to U22 and small RAEs in the U23. With a large OR of 3.1, RAEs were highest in the youngest age category (U18) and consistently/continuously decreases to small RAEs demonstrated by an OR of 1.8 in the U23 (Table 2).

Table 3 shows market values across each age group and all playing positions separated by birth quartile. In the U19 a small effect with an OR of 1.2 was found. The RAEs in the U18, U20, and U21 were negligible. A medium inverse effect (OR 0.5 [95%CI 0.4, 0.5]), where Q4 players had a higher market value, were found in the U22 and a small effect in the U23 (OR 0.7 [95%CI 0.6, 0.8]).

Table 4 shows the difference of observed and expected market values across each age group and Q. In Q1 and Q2 observed values were constantly higher than expected values. In contrast, in Q3 and Q4 observed values were constantly lower than expected. Within the age categories, there was a constant decrease in values from Q1 to Q4. In the overall group, this leads to a deviation/overestimation of €1.2 billion in Q1 and a deviation/underestimation of €1.4 billion in Q4.

Distribution of player positions per Q with 95% CI are illustrated in Table 5. There were medium to large RAEs in all positions from U18 to U22. The highest ORs were found in the U18 age category, except for goalkeepers. There were no significant differences between the different playing positions.

Table 6 displays position specific RAEs between Q1 and Q4 players for each age group based on market values. Market value was greater for relatively older goalkeepers (Q)1 compared to Q4, with a small to large effect depending on age group. The market values of defenders, midfielders and strikers were significantly higher for Q4 compared to Q1 players in the U21, U22 and U23 with small to large effects. Over- and undervaluing due to RAEs were highest for strikers, followed by defenders, midfielders, and goalkeepers.

## 4. Discussion

Results from the present study, illustrate the following main findings: (i) the analysis of relative age distribution illustrated significant overrepresentations of Q1 players in all age categories. Effect sizes diminished progressively from the U18 (large) to the U23 (small). This trend only existed when analyzing the whole sample, not when separated by playing positions. (ii) Relative age was also associated with biased market values. Initially, higher market values were apparent for Q1 players at U19. Thereafter, the effect was inversed, with Q4 players showing a significantly higher market value across U21, U22, and U23. (iii) Playing positions analysis revealed higher market values for Q4 defenders, midfielders, and strikers at U23, compared to Q1. By contrast, relatively older goalkeepers (Q1) had a higher market value than Q4 goalkeepers in all age categories.

Present findings align with previous studies, where RAEs biases were evident in the sample [2]. Biased selection during youth talent development programs may reduce a relatively younger athlete’s chances of succeeding later in their career. The relatively younger are disadvantaged by lower selection quotas, which in turn may lead to less competition experience, lower motivation, as well as a lower opportunities of accessing high-quality training [2]. However, particular RAE studies identify inverse RAEs in talent development programs post-puberty [9,12], suggesting delayed benefits if the relatively young can remain within the sporting development system. For instance, Deaner (2013) showed how compared to those born in Q1, Q3 and Q4 players were twice as likely to reach professional career benchmarks. Similarly, Fumarco (2017) identified how Q4 players scored more often, and receive higher salaries, than Q1 players. When considered alongside present findings, the underdog effect is supported, reflected by the increased likelihood of being drafted, career length, performance productivity, and now market value at the professional level [25].

The phenomenon that Q4 athletes are over-represented among those who successfully transition from youth systems to senior professional status has been called the ‘underdog hypothesis’. Being younger essentially facilitates long-term development by necessitating them to overcome the relative age disadvantage, through being challenged by their older and more advanced peers [10,11,12]. A previous study by Doyle and Bottomley (28), who analyzed the market values of the top 1000 players on transfermarkt.de in the season of 2013–2014, noted that relatively older players had greater opportunities due to assessment selection bias, but were valued equally to players born later in the year. Although the current study confirms these results, the market values of players do represent the underdog effect. As such, selected Q4 players are often initially undervalued, but later are valued higher than Q1 players [9]. Additionally, a recent study of Perez-Gonzalez et al. [26] analyzed the market value of 2577 adult professional players of the biggest European football leagues. Small to medium RAEs were shown in all leagues (*p* < 0.05). However, this bias did not affect the market value of the professional elite soccer players examined. The authors concluded that identification and promotion of talent at young ages are often biased by RAEs, however once players have reached the professional stage, their market value is independent of RAEs [26]. In our study, from a return of investment point of view, market value of Q1 players increases by 560% from U18 to U23, whereas market value of Q4 players increases by 810%. This phenomenon is even more pronounced when differentiated by playing position. The value of Q4 goalkeepers and defenders increases by approximately 3000%, while the value of Q1 players “only” increases by 1260% and 760%, respectively. In the U23, the highest mean values in terms of playing positions were found for defenders, midfielders and strikers born in Q4, except for goalkeepers. This leads to the assumption that the underdog effect exists as well if the sample is subdivided by playing positions. To sum up, RAEs and biased market values likely lead to inefficient selection and return of investment of football talent. To gain further insight into this issue, longitudinal studies analyzing the evolution of market values of players throughout talent development should be conducted.

Limitations: while the present analysis was performed using a cross sectional dataset, future studies should use a longitudinal design to analyze the evolution of market values and their interrelationships with RAEs. Furthermore, as financial loss due to over- and undervaluing was calculated on a theoretical estimate assuming an equal distribution of players between birth quarters, future studies which particularly focus on this aspect, should also include factors such as the evolution of market values in the long run, differences between female and male sports and the optimal talent development from a sports-scientific and economic point of view.

## 5. Conclusions

The analysis of relative age distribution illustrated significant overrepresentations of Q1 players in all age categories. This trend only existed when analyzing the whole sample, not when separated by playing positions. Relative age was also associated with biased market values. Initially, higher market values were apparent for Q1 players at U19. Thereafter, the effect was inversed, with Q4 players showing a significantly higher market value across U21, U22, and U23. Playing positions analysis revealed higher market values for Q4 defenders, midfielders, and strikers at U23, compared to Q1. By contrast, relatively older goalkeepers (Q1) had a higher market value than Q4 goalkeepers in all age categories. Assuming an equal distribution of football talent exists across annual cohorts, findings suggest the selection and market value of young professional players is dynamic. Findings suggest a potential biased selection, and undervaluing of Q4 players in younger age groups, as their representation and market value increased over time. By contrast, the changing representations and market values of Q1 players suggest initial overvaluing in performance and monetary terms.

## Figures and Tables

**Table 1:** Subject characteristics per age category.

under (U)	n	Age (Years)	Height (cm)	Market Value (€)	n (Clubs)	n (Countries)
18	1738	17.4 ± 0.5	180.0 ± 7.0	326,252 ± 1,878,569	941	98
19	2000	18.6 ± 0.3	180.6 ± 6.8	399,588 ± 1,957,796	1149	105
20	2000	19.6 ± 0.3	180.5 ± 7.0	853,200 ± 4,360,673	1140	110
21	2000	20.6 ± 0.3	180.6 ± 6.9	1,255,337 ± 4,761,941	1118	118
22	2000	21.6 ± 0.3	180.6 ± 6.8	1,367,525 ± 6,070,609	1119	119
23	2000	22.6 ± 0.3	180.8 ± 6.9	1,968,675 ± 5,863,952	1077	117
Total	11,738	20.1 ± 1.7	180.6 ± 6.9	1,043,561 ± 4,552,652	2861	153

Note: Data presented as mean ± the standard deviation or frequency (n).

**Table 2:** Distribution of players per age category and quarter (Q).

under (U)	n	Q1	Q2	Q3	Q4	OR Q1/Q4	95% CI	Effect Size
18	1738	705 (40.6%)	462 (26.6%)	340 (19.6%)	231 (13.3%)	3.1 *	(2.6, 3.6)	large
19	2000	746 (37.3%)	574 (28.7%)	402 (20.1%)	278 (13.9%)	2.7 *	(2.3, 3.1)	medium
20	2000	783 (39.2%)	509 (25.5%)	410 (20.5%)	298 (14.9%)	2.6 *	(2.3, 3.0)	medium
21	2000	722 (36.1%)	537 (26.9%)	439 (22%)	302 (15.1%)	2.4 *	(2.1, 2.8)	medium
22	2000	700 (35%)	560 (28%)	417 (20.9%)	323 (16.2%)	2.2 *	(1.9, 2.5)	medium
23	2000	659 (33%)	531 (26.6%)	452 (22.6%)	358 (17.9%)	1.8 *	(1.6, 2.1)	small
Total	11,738	4315 (36.8%)	3173 (27%)	2460 (21%)	1790 (15.2%)	2.4 *	(2.2, 2.6)	medium

Note: RAEs of players listed in Tranfermarkt.de. Q1 to Q4 = Quartile 1 to 4; OR = Odds ratio; CI = Confidence Interval; * *p* < 0.05; OR < 1.22, 1.22 ≤ OR < 1.86, 1.86 ≤ OR < 3.00, and OR ≥ 3.00, was interpreted as negligible, small, medium and large.

**Table 3:** Mean market values per age category and relative age quartile (Q).

under (U)	n	Q1 (€)	Q2 (€)	Q3 (€)	Q4 (€)	OR Q1/Q4	95% CI	Effect Size
18	1738	318,950	320,963	328,971	327,597	1.0	(0.9, 1.0)	no
19	2000	469,437	373,563	317,910	383,993	1.2 *	(1.1, 1.3)	small
20	2000	942,593	711,690	809,939	919,547	1.0	(1.0, 1.0)	non
21	2000	1,183,587	1,311,778	1,252,790	1,330,215	0.9 *	(0.9, 0.9)	non
22	2000	1,136,464	1,285,982	1,052,338	2,416,563	0.5 *	(0.5, 0.5)	medium
23	2000	1,789,416	2,112,712	1,519,967	2,651,536	0.7 *	(0.7, 0.7)	small
Total	11,738	960,002	1,031,007	913,638	1,445,796	0.7 *	(0.6, 0.7)	small

Note: Q1 to Q4 = Quartile 1 to 4; OR = Odds Ratio; CI = Confidence Interval; * *p* < 0.05; 1.00 ≤ OR < 1.22, 1.22 ≤ OR < 1.86, 1.86 ≤ OR < 3.00, and OR ≥ 3.00, was interpreted as negligible, small, medium and large. Inverse ORs < 0.33 (1/3), 0.33 ≤ OR < 0.53 (1/1.86), 0.53 ≤ OR < 0.81, 0.81 ≤ OR < 1.0 were interpreted as large, medium, small and negligible.

**Table 4:** Δ of total market values per age category and relative age quartile (Q).

under (U)	n	Δ Q1 (€)	Δ Q2 (€)	Δ Q3 (€)	Δ Q4 (€)
18	1738	86,276,071	8,826,488	−31,087,721	−66,666,071
19	2000	115,481,501	27,643,641	−31,155,224	−85,246,403
20	2000	266,753,704	6,405,206	−72,894,512	−185,748,490
21	2000	262,756,371	48,535,801	−76,420,216	−263,382,616
22	2000	227,292,857	77,158,929	−87,344,065	−427,731,734
23	2000	284,517,109	65,494,068	−72,958,407	−376,518,156
Total	11,738	1,243,077,614	234,064,133	−371,860,145	−1,405,293,470

Note: Difference of observed and expected market values (Δ). Q1 to Q4 = Quartile 1 to 4.

**Table 5:** Distribution of player positions per age category and quarter (Q).

Position	under (U)	n	Q1 (%)	Q2 (%)	Q3 (%)	Q4 (%)	OR Q1/Q4	95% CI	Effect Size
Goalkeeper	18	240	89 (37.1)	65 (27.1)	49 (20.4)	37 (15.4)	2.4 *	(2.3, 2.5)	medium
	19	191	67 (35.1)	52 (27.2)	44 (23.0)	28 (14.7)	2.4 *	(2.3, 2.5)	medium
	20	149	62 (41.6)	38 (25.5)	30 (20.1)	19 (12.8)	3.3 *	(3.1, 3.4)	large
	21	144	55 (38.2)	34 (23.6)	27 (18.8)	28 (19.4)	2.0 *	(1.9, 2.1)	medium
	22	109	43 (39.4)	31 (28.4)	23 (21.1)	12 (11.0)	3.6 *	(3.4, 3.7)	large
	23	114	39 (34.2)	35 (30.7)	21 (18.4)	19 (16.7)	2.1 *	(2.0, 2.1)	medium
Defender	18	431	212 (49.2)	114 (26.5)	89 (20.6)	50 (11.6)	4.2 *	(4.0, 4.4)	large
	19	558	226 (40.5)	161 (28.9)	101 (18.1)	70 (12.5)	3.2 *	(3.1, 3.4)	large
	20	560	219 (39.1)	143 (25.5)	116 (20.7)	82 (14.6)	2.7 *	(2.6, 2.8)	medium
	21	609	230 (37.8)	165 (27.1)	120 (19.7)	94 (15.4)	2.4 *	(2.4, 2.6)	medium
	22	639	224 (35.1)	169 (26.4)	136 (21.3)	110 (17.2)	2.0 *	(2.0, 2.1)	medium
	23	676	238 (35.2)	172 (25.4)	144 (21.3)	122 (18)	2.0 *	(1.9, 2.0)	medium
Midfielder	18	606	236 (38.9)	164 (27.1)	119 (19.6)	87 (14.4)	2.7 *	(2.6, 2.8)	medium
	19	711	255 (35.9)	218 (30.7)	141 (19.8)	97 (13.6)	2.6 *	(2.5, 2.7)	medium
	20	713	272 (38.1)	183 (25.7)	144 (20.2)	114 (16.0)	2.4 *	(2.3, 2.5)	medium
	21	681	243 (35.7)	173 (25.4)	171 (25.1)	94 (13.8)	2.6 *	(2.5, 2.7)	medium
	22	669	250 (37.4)	204 (30.5)	120 (17.9)	95 (14.2)	2.6 *	(2.5, 2.7)	medium
	23	627	179 (28.5)	179 (28.5)	155 (24.7)	114 (18.2)	1.6 *	(1.5, 1.6)	small
Striker	18	461	202 (43.8)	119 (25.8)	83 (18)	57 (12.4)	3.5 *	(3.4, 3.7)	large
	19	540	198 (36.7)	143 (26.5)	116 (21.5)	83 (15.4)	2.4 *	(2.3, 2.5)	medium
	20	578	230 (39.8)	145 (25.1)	120 (20.8)	83 (14.4)	2.8 *	(2.7, 2.9)	medium
	21	566	194 (34.3)	165 (29.2)	121 (21.4)	86 (15.2)	2.3 *	(2.2, 2.4)	medium
	22	583	183 (31.4)	156 (26.8)	138 (23.7)	106 (18.2)	1.7 *	(1.6, 1.8)	small
	23	583	203 (34.8)	145 (24.9)	132 (22.6)	103 (17.7)	2.0 *	(1.9, 2.1)	medium

Note: Q1 to Q4 = Quartile 1 to 4; OR = Odds Ratio; CI = Confidence Interval; * *p* < 0.05; 1.00 ≤ OR < 1.22, 1.22 ≤ OR < 1.86, 1.86 ≤ OR < 3.00, and OR ≥ 3.00, was interpreted as negligible, small, medium and large.

**Table 6:** Market values per playing position, age category and relative age quartile (Q).

Position	under (U)	n	Q1 (€)	Q2 (€)	Q3 (€)	Q4 (€)	OR Q1/Q4	95% CI	Effect Size
Goalkeeper	18	240	159,238	140,860	33,724	24,024	6.6 *	(6.4, 6.9)	large
	19	191	107,090	90,385	101,705	83,929	1.3 *	(1.2, 1.3)	small
	20	149	318,548	265,789	251,667	236,842	1.3 *	(1.3, 1.4)	small
	21	144	1,750,909	217,647	432,407	413,393	4.2 *	(4.0, 4.4)	large
	22	109	786,628	1,112,903	370,652	581,250	1.4 *	(1.3, 1.4)	small
	23	114	2,008,333	1,289,286	1,257,143	707,895	2.8 *	(2.7, 3.0)	medium
Defender	18	431	192,191	265,570	114,326	74,500	2.6 *	(2.5, 2.7)	medium
	19	558	473,341	385,093	135,891	261,786	1.8 *	(1.7, 1.9)	medium
	20	560	689,954	455,944	560,129	1,484,146	0.5 *	(0.5, 0.5)	medium
	21	609	1,016,739	1,233,636	1,392,292	1,347,340	0.8 *	(0.7, 0.8)	small
	22	639	1,221,205	999,260	767,463	2,872,045	0.4 *	(0.4, 0.4)	medium
	23	676	1,457,248	1,458,285	1,083,854	2,216,189	0.7 *	(0.6, 0.7)	small
Midfielder	18	606	501,907	334,146	271,639	245,690	2.0 *	(2.0, 2.1)	medium
	19	711	288,431	329,128	324,823	177,835	1.6 *	(1.6, 1.7)	small
	20	713	694,210	877,869	479,688	664,035	1.0 *	(1,0, 1.1)	non
	21	681	1,331,173	1,533,671	1,350,585	615,160	2.2 *	(2.08, 2.3)	medium
	22	669	1,311,600	1,616,299	1,373,958	1,462,895	0.9 *	(0.9, 0.9)	non
	23	627	2,199,581	2,425,279	1,407,903	2,472,368	0.9 *	(0.9, 0.9)	non
Striker	18	461	318,688	492,731	808,735	863,158	0.4 *	(0.4, 0.4)	medium
	19	540	820,707	531,294	550,000	829,217	1.0	(1.0, 1.0)	non
	20	578	1,645,109	871,034	1,587,292	868,976	1.9 *	(1.8, 2.0)	medium
	21	566	1,035,696	1,382,727	1,159,298	2,391,570	0.4 *	(0.4, 0.5)	medium
	22	583	875,683	1,199,038	1,167,029	3,006,368	0.3 *	(0.3, 0.3)	large
	23	583	1,775,123	2,701,897	2,169,129	3,724,029	0.5 *	(0.5, 0.5)	medium

Note: Difference of observed and expected market values (Δ). Q1 to Q4 = Quartile 1 to 4; OR = Odds Ratio; CI = Confidence Interval; * *p* < 0.05; 1.00 ≤ OR < 1.22, 1.22 ≤ OR < 1.86, 1.86 ≤ OR < 3.00, and OR ≥ 3.00, was interpreted as negligible, small, medium and large. Inverse ORs < 0.33 (1/3), 0.33 ≤ OR < 0.53 (1/1.86), 0.53 ≤ OR < 0.81, 0.81 ≤ OR < 1.0 were interpreted as large, medium, small and negligible.

## Data Availability

All data used in this study are available on the website www.transfermarkt.de (accessed on 17 July 2020).
